# Single-cell transcriptomics identifies a dampened neutrophil function and accentuated T-cell cytotoxicity in tobacco flavored e-cigarette exposed mouse lungs

**DOI:** 10.1101/2025.02.17.638715

**Published:** 2025-02-22

**Authors:** G Kaur, T Lamb, A Tjitropranoto, Irfan Rahman

## Abstract

E-cigarettes (e-cigs) are a public health concern for young adults due to their popularity and evidence for increased oxidative stress and immunotoxicity. Yet an extensive study defining the cell-specific immune changes upon exposure to flavored e-cigs remains elusive. To understand the immunological lung landscape upon acute nose-only exposure of C57BL/6J to flavored e-cig aerosols we performed single-cell RNA sequencing (scRNA seq). scRNA profiles of 71,725 cells were generated from control and treatment groups (n=2/sex/group). A distinct phenotype of Ly6G-neutrophils was identified in lungs exposed to tobacco flavored e-cig aerosol which demonstrated dampened IL-1 mediated and pattern recognition signaling as compared to air controls. Differential gene expression analyses identified dysregulation of T-cell mediated pro-inflammation ( *Cct7* , *Cct8* ) and stress-response signals ( *Neurl3* , *Stap1* , *Cirbp* and *Htr2c)* in the lungs of mice exposed to e-cig aerosols, with pronounced effects for tobacco flavor. Flow cytometry analyses and cytokine/chemokine assessments within the lungs corroborated the scRNA seq data, demonstrating a significant increase in T-cell percentages and levels of T-cell associated cytokine/chemokines in the lungs of tobacco-flavored aerosol exposed mice. Increased levels of *Klra4* and *Klra8* expression also suggest an enhanced natural killer (NK) cell activity in this mouse group. Overall, this is a pilot study identifying increase in the percentages of Ly6G-neutrophils that may be responsible for dampened innate immune responses and heightened T-cell cytotoxicity in lungs of tobacco-flavored e-cig aerosol exposed mice. In addition, we provide preliminary evidence for sex-specific changes in the transcriptional landscape of mouse lungs upon exposure to e-cig aerosol, an area that warrants further study.

## Full Text Availability

The license terms selected by the author(s) for this preprint version do not permit archiving in PMC. The full text is available from the preprint server.

